# Developmental Perchlorate Exposure and Synaptic Transmission in Hippocampus

**DOI:** 10.1289/ehp.0800532

**Published:** 2009-06

**Authors:** Richard D. Mavis, John M. DeSesso

**Affiliations:** Noblis Falls Church, Virginia E-mail: rmavis@noblis.org

In “Developmental Exposure to Perchlorate Alters Synaptic Transmission in Hippocampus of the Adult Rat,” Gilbert and Sui (2008) reported results of exposure of pregnant rat dams to perchlorate in drinking water, with the purpose of evaluating neurologic development in rat pups after *in utero* exposure to perchlorate.

We cannot agree with the authors’ conclusion that their findings

indicate that neurologic impairment is associated with modest degrees of thyroid hormone insufficiency and support previous animal studies of neurodevelopmental sequelae associated with low levels of perchlorate exposure.

Also, we do not agree that their data (Gilbert and Sui 2008) “provide evidence in a rodent model that modest degrees of thyroid hormone reduction induced by perchlorate result in persistent decrements in brain function.”

The “association” of the electro physiologic anomalies measured in the hippocampus [interpreted by Gilbert and Sui (2008) as “neurologic impairment” or “persistent decrements in brain function”] with “modest degrees of thyroid hormone insufficiency” in the pups is not, in our opinion, a credible interpretation of the authors’ results, because the evidence for “thyroid hormone insufficiency” is questionable, the electrophysiologic anomalies did not demonstrate a consistent dose–response relationship, and behavioral tests, chosen for their sensitivity to hippocampal deficiencies, did not show any behavioral changes associated with the measured electro physiologic anomalies.

Pregnant rat dams were exposed to perchlorate in drinking water in four experimental dose groups (0, 30, 300, and 1,000 ppm) from gestational day 6 until pups were weaned on postnatal day (PND) 30. The active thyroid hormone triiodothyronine (T_3_), its inactive precursor thyroxine (T_4_), and thyroid-stimulating hormone (TSH) were measured in the serum of rat pups on PNDs 4, 14, and 21. Hormonal levels were not affected on PNDs 4 or 14, with the exception of a marginal but statistically significant increase in serum TSH on PND14 in the two lower dose groups (30 and 300 ppm perchlorate). The statistical significance of these results is questionable because changes of similar or greater magnitude in serum TSH on PND14 in the highest dose group (1,000 ppm) were not statistically significant.

On PND21, serum T_3_ was reduced approximately 10–14% in the two higher dose groups (Gilbert and Sui 2008). These changes are similar to the intra- and inter-assay variations in these measure ments, stated in the “Methods” to be 9–12%. Serum T_4_ at PND21 was reduced by approximately 11% in the 300-ppm dose group and 27% in the 1,000-ppm dose group. The authors observed no statistically significant change in TSH in any of the dose groups on PND21.

Thus, in three dose groups at three time points for three serum thyroid hormones—a total of 27 data points—Gilbert and Sui (2008) found no changes in the active serum thyroid hormone T_3_ that were greater than the intra- and inter assay variations. Only 3 of 27 possible data points showed changes in any of the serum thyroid hormones: TSH at PND14 increased marginally in the two lower dose groups (but not in the highest dose group), and T_4_ (the precursor to the active hormone T_3_) decreased by 27% on PND21 in the highest dose group.

Because Gilbert and Sui (2008) found no changes in the active thyroid hormone T_3_ and questionable changes in other serum thyroid hormones measured in rat pups, we cannot agree with their interpretation that these data denote “thyroid hormone insufficiency” or “thyroid hormone reduction.”

The implication by Gilbert and Sui (2008) that the development of the hippocampus in rat pups is impaired as a result of perchlorate exposure of pregnant dams is weakened by the lack of consistent dose responses in the electrophysiologic parameters measured in the hippocampus of the rat pups. For example, the essentially identical curves for the 30- and 300-ppm dose groups shown in their Figure 4B, and the statistical equivalence of these curves with the control curve, leads us to question the relationship of these changes to perchlorate dosage. In Figure 5, the lack of dose dependence is evident as the changes in 300-ppm dose values often exceed or equal the changes in the 1,000-ppm dose values. The apparently random nature of the results of the various electrophysiologic tests used undermines any claim of reproducibility of their findings.

Interpretation of the reported electrophysiologic anomalies in the hippocampus as “neurologic impairment” or “decrements in brain function” by Gilbert and Sui (2008) was not supported by the results of behavioral testing. The authors chose four different behavioral tests for motor activity, spatial learning, and fear conditioning because of their sensitivity to hippocampal deficiencies. Rat pups in the three experimental dose groups performed equivalently to those in the control (unexposed) group on all behavioral tests. Thus, none of these tests demonstrated any “neurologic impairment” or *“*decrements in brain function.”

We also wish to comment on the relevance of this study (Gilbert and Sui 2008) to human health risk assessment. The perchlorate concentrations in drinking water the authors used (30–1,000 ppm) are 2–3 orders of magnitude higher than environmental concentrations, which range up to a maximum of 200 ppb in the United States (National Research Council 2005). The lack of biologically functional effects observed at the 30-ppm drinking water dose (Gilbert and Sui 2008) indicate that the environmental concentrations found in the United States have a 150-fold margin (30 ppm ÷ 200 ppb) of safety against any effects suggested by these authors, based on water concentrations alone. Considering that rats consume approximately 5 times more water per kilogram body weight than humans would increase the margin of safety to 600-fold (5 × 150).

In addition to the shortcomings of the study noted above, the rat is questionable as a model for the sensitivity of the human thyroid system to perchlorate because of differences in thyroid hormone storage. Humans are less sensitive than rats to inhibition of thyroid hormone synthesis by perchlorate because humans are capable of storing several months supply of sequestered T_4_ and T_3_, whereas rats are capable of storing only a few days supply of these hormones. Given the inadequacies of the experimental model, the absence of a dose response in the findings, and lack of corroboration of alleged hippocampal deficiency by behavioral tests, we believe that Gilbert and Sui’s (2008) conclusions are not substantiated.
